# Dietary Fibers and Cardiometabolic Diseases

**DOI:** 10.3390/ijms13021524

**Published:** 2012-02-02

**Authors:** Graziano Riccioni, Valeriana Sblendorio, Eugenio Gemello, Barbara Di Bello, Luca Scotti, Salvatore Cusenza, Nicolantonio D’Orazio

**Affiliations:** 1 Cardiology Unit, San Camillo de Lellis Hospital, Manfredonia, Foggia, 71043, Italy; 2 Human Nutrition, Department of Biochemical Sciences, University “G. D’Annunzio” Chieti, 66013, Italy; E-Mails: egemello@libero.it (E.G.); dibello.barbara@yahoo.it (B.D.B.); luca_scotti@yahoo.it (L.S.); qusenza@libero.it (S.C.); ndorazio@unich.it (N.D.); 3 Department of General Surgery and Surgical Specialties, University of Modena and Reggio Emilia Medical School, Surgical Clinic, Modena, 41100, Italy; E-Mail: valsbl@hotmail.it

**Keywords:** dietary fiber (DF), polyunsaturated fatty acids (PUFAs), cardiovascular disease (CVD)

## Abstract

The high prevalence of cardiovascular disease (CVD) is largely attributable to the contemporary lifestyle that is often sedentary and includes a diet high in saturated fats and sugars and low ingestion of polyunsaturated fatty acids (PUFAs), fruit, vegetables, and fiber. Experimental data from both animals and humans suggest an association between increased dietary fiber (DF) intakes and improved plasma lipid profiles, including reduced low density lipoprotein cholesterol (LDL-C) concentrations. These observations underline that the intake of DF may protect against heart disease and stroke.

## 1. Introduction

Dietary fibers (DF) are highly complex substances described as nondigestible carbohydrates and lignins resistant to digestion and absorption in the small intestine [1]. Commonly, DF is classified according to solubility in water, even though grading according to viscosity, gel-forming capabilities, or fermentation rate by the gut flora might be physiologically more relevant [2]. Indeed the National Academy of Sciences Panel for the definition of DF recommended that the terms soluble and insoluble should be gradually replaced by terms referring to the specific beneficial physiological effects associated with the fiber: viscosity and fermentability [3].

Fruit, vegetables, wholegrains, and cereals are the major sources of DF components. Total DF can be divided into two groups: viscous fiber (pectin, gums, and mucilage, which were previously classified as water-soluble fiber) and non-viscous fiber (cellulose, hemicellulose, and lignin, which were previously classified as water-insoluble fiber).

DF are indigestible substances resistant to human digestive enzymes without nutritional or energetic value (except for the small amount of energy derived from short chain fatty acids such as acetate, propionate and butyrate produced by the bacterial flora of the colon). DF was an almost unknown phrase and fibers were considered only annoying intestinal wastes until the 1970s when a wide range of potential therapeutic applications were suggested [4].

The high prevalence of cardiovascular disease (CVD) is largely attributable to the contemporary sedentary lifestyle combined with a diet high in saturated fats and sugars, and low ingestion of polyunsaturated fatty acids (PUFAs), fruit, vegetables, and fiber. Epidemiological studies have confirmed a strong association between fat intake, especially saturated- and transfatty acids, plasma cholesterol levels, and rate of coronary heart disease (CHD) mortality [5]. In contrast, beneficial cardiovascular effects have been reported in populations consuming the “healthy” Mediterranean-type diet [6]. Indeed, many nutrients and phytochemicals in fruits, vegetables, and wine, including fiber, vitamins, minerals, antioxidants, have shown to be independently or jointly responsible for the apparent reduction in CVD risk [7]. Undoubtedly, the advances in the knowledge of both the disease processes and healthy DF components have provided new avenues to develop dietary strategies to halt the development of CVD. In this regard, a growing body of clinical evidence has demonstrated positive cardiovascular effects associated with DF intake [8].

## 2. Biochemistry of the Fibers

The term DF includes a wide range of molecular structures, a highly complex mixture of different non-starch polysaccharides (NSPs), which include cellulose, β-glucans, hemicellulose, pectins, gums, polysaccharides of algae (agar and carrageenan) and lignin (polymers of phenylpropane, found in most plant structures associated with cellulose) [9,10]. Sources of DF can be classified as providing soluble or insoluble fiber, based on dispersibility of the polysaccharide in water, rather than on true chemical solubility [11].

The two major classes of DF are polysaccharides and lignin. Polymers of phenylpropane, are found in most plant structures in association with cellulose, and is the most widespread organic molecule on Earth and the major component of plant cell walls. It is a linear polymer made up of 10,000 to 15,000 glucose molecules bonded in a 1→4 glycosidic linkage. Cellulose molecules contain many polar hydroxyl groups, which allow them to interact with adjacent molecules to form fibers. These fibers are structurally strong and resistant to chemical attack. The more important non-cellulosic polysaccharide (NCPs) is hemicellulose and pectic substance. Hemicellulose are cell wall polysaccharides solubilized by aqueous alkali, which contain backbones of β-1,4-linked pyranoside sugar, and differ from cellulose in size (less than 200 sugar residues). The hemicelluloses are subclassified on the basis of the principal monomeric sugar residue [12]. Pectin substances are a complex group of polysaccharides in which d-galacturonic acid is a principal constituent. They are structural component of plant cell walls and also act as intercellular cementing substances. The backbone structure of pectin is an unbranched chain of axial-axialα-(1-4)-linked d-galacturonic acid units. Pectin is highly water soluble and is almost completely metabolized by colonic bacteria. Other NCPs include gums, mucillages, and algal polysaccharides. Lignin is a cross-linked racemic macromolecule, a three-dimensional aromatic polymer of phenylpropane derivatives (containing about 40 oxygenated phenylpropane unit), and is associated with cellulose in plant cell walls. Lignins vary in molecular weight and methoxyl content. Due to strong intramolecular bonding which includes carbon to carbon linkages, lignin is very inert [13].

The soluble DF has strong probiotic characteristics, which can be considered a non-digestible food ingredient that can selectively stimulate the growth and metabolic activity of a limited number of microbial groups, important for the proper functioning of the body. Recently, there has been a further substantial revision of the view of what exactly constitutes DF with the emerging recognition of the contribution of resistant starch and oligosaccharides to ‘fiber’ action [14]. It was thought that all of dietary starch consumed in cooked foods was digested in the human small intestine. It is becoming clear that this is not so, and that a substantial fraction of ingested starch, resistant starch, escapes from the small intestine and enters the colon of healthy humans. In terms of the dietary polysaccharides entering that viscous, resistant starch may exceed NSPs in quantity and in the range of its actions. In the large bowel, NSPs and resistant starch are fermented by the microflora, yielding metabolic end products, which may mediate some of the health benefits ascribed to the carbohydrates. Undigested protein (resistant protein) and other non-digested carbohydrates also contribute to large bowel fermentation. These non-digested fractions contribute to DF via fermentation and could be considered in net dietary fiber intake.

## 3. Fiber and Cardiovascular Disease

Consumption of DF has been associated to lower risk of CVD for some time [15], but the hypothesis that DF intake could protect directly against CVD is relatively recent [16–22]. Experimental data from both animals and humans suggest an association between increased DF intakes and improved plasma lipid profiles, including reduced low density lipoprotein cholesterol (LDL-C) concentrations. These observations indicated a regulation pathway between fiber, plasma lipids, and atherosclerosis [23].

Soluble fiber clearly lowers cholesterol to a small but significant degree and one would expect that this would reduce coronary heart disease (CHD) events [24]. The specific effects of food structure (increased satiety, reduced transit time and glycaemic response), together with the antioxidant and anti-carcinogenic properties of numerous bioactive compounds, especially those in the bran and germ (minerals, trace elements, vitamins, carotenoids, polyphenols and alkylresorcinols), are today well-recognized mechanisms. Recent findings, the exhaustive listing of bioactive compounds found in whole-grain wheat, their content in whole-grain, bran and germ fractions and their estimated bioavailability, have led to new hypotheses. Whole-grain wheat is also a rich source of methyl donors and lipotropes (methionine, betaine, choline, inositol and folates) that may be involved in cardiovascular and/or hepatic protection, lipid metabolism and DNA methylation [25].

### 3.1. Fiber and Cardiovascular Disease: Epidemiological Studies

Many epidemiological studies examined the relationship between wholegrain consumption and CHD. In these studies the researchers concluded that a relationship between wholegrain intake and CHD is seen with at least a 20% and perhaps a 40% reduction in risk for those who eat wholegrain food habitually *vs*. those who eat them rarely [26]. Notwithstanding the fact that fiber is an important component of wholegrains, many studies have not shown an independent effect of fiber alone on CHD events [27].

A study about the relationship of long-term intake of dietary fiber by 68,782 women showed a substantial lowering of relative risk (0.53) for women in the highest quintile of fiber consumption (22.9 g/day) compared with the lowest (11.5 g/day). These intakes are low compared to those recommended by health authorities (30 g/day). Only the effect of cereal fiber was significant. However, the question of the relationship of other contributors to the effects of DF (e.g., resistant starch) and CHD risk remains unanswered. Elevated plasma total and low-density lipoprotein cholesterol (LDL-C) concentrations are established risk factors for coronary morbidity and mortality. There are abundant human and animal data showing that diets high in soluble fiber lower plasma cholesterol [28]. One population study has shown a significant negative relationship between viscous (soluble) fiber intake and carotid artery atherogenesis as measured by carotid intima–media thickness (CIMT). This association was significant statistically even though average fiber intakes were not particularly high [29–30]. A combined analysis of ten prospective cohort studies conducted in the USA and Europe showed a 25% decrease in the risk of CHD for each 10 grams increase in fiber intake, after adjusting for several dietary and cardiovascular confounding factors [31]. Recently, Streppel *et al.* [32] observed that CHD mortality and all-cause mortality were reduced by 17% and 9% respectively for every additional 10 g of dietary fiber/day, with no clear associations for different types of DF. The most likely direct protective role for DF in CHD etiology is through plasma lipid lowering. The effect appears to be specific for plasma total and LDL-C, and, possibly, triacylglycerols (TAG). Of the main fiber components, soluble NSPs seem to be effective, but insoluble NSPs and resistant starch are not. This paradox can be explained by the fact that food rich in soluble fiber contains other phytochemical compounds that have been demonstrated to modulate inflammation, oxidation, insulin resistance and cholesterol metabolism. Likewise, it is possible that the effects attributed to fiber intake may instead be an indicator of lifestyle and of a healthy dietary pattern. There is good evidence from animal and human studies to support a hypocholesterolemic effect of soluble NSPs either in enriched plant fractions (e.g., oat bran) or as natural (e.g., pectins and guar gum) or synthetic isolates (e.g., hydroxypropylmethylcellulose). The magnitude of the effect varies with dose. There are several hypotheses to explain the NSP action on plasma cholesterol, including enhanced bile acid and neutral sterol excretion, the slowing of fat and cholesterol absorption and direct inhibition of hepatic cholesterol synthesis by propionate formed by large bowel fermentation of NSPs [33,34]. Any increase in bile acid excretion leads to enhanced hepatic uptake of cholesterol and its conversion to bile acids with a consequent depletion of the plasma cholesterol pool [35]. It was initially thought that fiber could bind some bile acids selectively, in a similar manner to cholestyramine, an ion exchange resin that binds bile acids; but cholestyramine is strongly charged, whereas most NSPs with cholesterol-lowering potential are neutral or even acidic (e.g., pectins). Neutrality is not consistent with ionic binding and uronic acid residues would repel bile acids at the pH of the small intestine. The property that appears to mediate the increased steroid excretion is the viscosity in solution. Presumably, bile acids are lost from the ileum through a form of entrapment in a viscous gel [36]. This would also contribute to the loss of cholesterol and the slower digestion of fat seen with ingestion of NSPs. Abundant animal and human data show that feeding soluble NSPs increases fecal steroid excretion. In addition, it is possible that other components in, or associated with, fiber (phytoestrogens or antioxidants) may be responsible for any observed protective effect. In fact some studies suggest that cardiovascular benefits are, to a great extent, a direct consequence of eating whole-grain bread, green-leafy vegetables, fruit and vegetables, more than dietary fiber itself [36,37].

An analysis of dietary factors and cardiovascular risk performed in a sample of 3452 Swiss adults demonstrate that a healthy diet characterized by high consumption of dietary fiber was associated with lower rates of serum triglycerides and higher values of high density lipoprotein cholesterol (HDL-C) [38]. An improvement in lipid profiles associated with high fiber consumption has also been observed in epidemiologic studies carried out on adults in Germany, China, Denmark, France, Greece, Italy and Maryland [39].

The American Heart Association (AHA) recommends a total dietary fiber intake of 25 g/day to 30 g/day from foods (not supplements) to ensure nutrient adequacy and maximize the cholesterol-lowering impact of a fat-modified diet [40]. Finally, as has been mentioned, both the American Dietetic Association (ADA) and the Institute of Medicine advise an intake of 14 g of dietary fiber per 1000 kcal, or 25 g/day for adult women and 38 g/day for adult men, to protect against CVD, and that these fibers should come from high-fiber foods [41].

### 3.2. Wholegrains and Coronary Heart Disease (CHD)

Wholegrain consumption has been shown to be linked to improvements in body mass index (BMI) [42,43] insulin sensitivity [44] and diabetes [45], all of which are important risk factors for CHD. Consumption of wholegrains has been shown to improve insulin sensitivity [46] although Juntunen *et al.* [47] did not confirm this. Several reviews published since 1999 have concluded that nutritional approaches to the prevention of CHD should include the consumption of wholegrains [48–52]. The studies reviewed defined wholegrain as either intact or milled grain with bran, germ and endosperm in the same proportion as the unmilled grain. Wholegrain foods were arbitrarily defined as those foods with 425% by weight wholegrain or bran. Anderson *et al*. [53] carried out a meta-analysis of results from three large prospective studies [54–56] which specifically examined the relationship of risk of CHD to wholegrain. They concluded that persons in the highest, compared to those in the lowest, quintile for wholegrain intake had a 28% reduction in their risk for CHD (RR 0.72, 95% CI, 0.49–0.94), a result which provides strong support for the hypothesis that generous intakes of wholegrains are cardioprotective. Jensen *et al.* [57] followed a cohort of 42,850 male health professionals aged 40–75 years who were free from CVD cardiov, cancer and diabetes at baseline in 1986. Daily wholegrain intakes (grams per day) were derived from a detailed and validated semiquantitative food frequency questionnaire (FFQ). The participants were followed with repeated questionnaires on lifestyle and health every 2 years and with detailed FFQs every 4 years. During 14 years of follow-up, 1818 incident cases of CHD occurred. After CHD risk factors and intakes of bran and germ added to foods were controlled for, the hazard ratio (HR) of CHD between lowest and highest quintiles of wholegrain consumption (median intake 3.5 *vs*. 42.4 g/day) was 0.82 (95% CI, 0.70–0.96). The authors acknowledge that a greater intake of wholegrains was related to an overall healthier diet and lifestyle. They performed stratified analyses to address potential confounding by healthy participant characteristics and found no discrepant results with respect to the main finding of protection from heart disease with wholegrains in subgroups including never smokers and non-drinkers.

A further review has also reported the results of more recent prospective studies of wholegrain consumption The reviewers concluded that a relationship between wholegrain intake and CHD is seen with at least a 20% and perhaps a 40% reduction in risk for those who eat wholegrain food habitually *vs.* those who eat them rarely [58].

Jacobs and Gallaher consider that, given the wide variability in study designs, an estimated risk reduction of 20–40% is ‘impressively robust’. Study participants included men and women; participants from the US and Norway. Findings, which were consistently positive for wholegrains, occurred using a variety of different data collection methodologies.

In conclusion, Jacobs and Gallaher and other reviewers find that there is good evidence that wholegrain foods substantially reduce the risk of CHD. Whether all grains are equal in this respect cannot be concluded from these studies, nor can the effectiveness of different parts of the grain. Results from Jensen *et al*. [57] suggest that added bran may confer additional benefits; clearly this proposition needs more verification.

### 3.3. Wholegrains and Stroke

Liu *et al.* [59] examined the relationship of wholegrain intake and risk of ischaemic stroke in a 12-year follow-up of the Nurses’ Health Study. The baseline population for analysis consisted of 75,521 women between (aged 38–63 years), without previous diagnosis of diabetes mellitus, angina, myocardial infarction, stroke or other CVD. Incident cases of stroke were confirmed by reviewing medical records and classified as ischemic where the stroke emanated from thrombotic or embolic occlusion of a cerebral artery, resulting in infarction. Wholegrain intake was reported through a self-administered FFQ. During the follow-up, an inverse association between wholegrain intake and ischaemic stroke risk was observed; the relative risk for ischemic stroke in the highest quintile was 0.69 (95% CI, 0.50–0.98). No such inverse relationship was observed for refined grain intake. The authors also examined the possibility that specific constituents (folates, potassium, magnesium, vitamin E and fiber) explained the protective effect of wholegrains. Adjustment for those components attenuated the inverse relation of wholegrain intake with ischemic stroke (RR 0.76; 95% CI, 0.51–1.15, when comparing the highest *vs* the lowest quintile). However, the inverse relation remained (even though not statistically significant) suggesting that other constituents may confer additional protection. The authors acknowledge that their findings may not be generalizable to other populations (98% of their cohort were white).

Steffen *et al*. [43] did include more African American men and women in their prospective study. They followed 11,940 men and women from four US cities, aged 45–64 years, over 11 years. Usual dietary intakes were assessed using a FFQ at baseline and 6 years later. Overall, they found a beneficial effect of wholegrain on risks of total mortality and incident CAD but not on the risk of ischaemic stroke, after adjustment for potential confounders (RR, 0.75, 95% CI, 0.46–1.22 when comparing the highest *vs* the lowest quintiles). Mozaffarian *et al*. [60] followed 3588 men and women aged 65 years at baseline for 8.6 years to determine the association between fiber consumption from fruit, vegetables and cereal sources and incident cerebrovascular disease, defined as combined incident stroke, fatal and nonfatal myocardial infarction, and CHD death. In secondary analyses, they found that higher cereal fiber intake was associated with lower risk of total stroke (HR 0.78, 95% CI, 0.64–0.95) and ischemic stroke (HR 0.76, 95% CI, 0.60–0.95) when comparing the 80th percentile with the 20th percentile.

Fung *et al.* [61] examined the relationship between stroke risks in women and overall dietary patterns, specifically a ‘prudent’ diet (characterized by higher intakes of fruits, vegetables, wholegrains, fish and poultry) and a ‘western’ diet (with higher intakes of red and processed meats, refined grains, full-fat dairy products, desserts and sweets). They followed 71,768 women aged between 38 and 63 years, without a history of CVD or diabetes at baseline, for 14 years. Dietary intake was ascertained by FFQ, which assessed food intake during the previous year, from which dietary pattern scores were calculated. After adjustment for stroke risk factors, they found women in the highest quintile of the Western pattern score had a relative risk of 1.58 (95% CI, 1.15–2.15) for total strokes and 1.56 (95% CI, 1.05–2.33) for ischemic stroke when compared with the lowest quintiles. For the prudent pattern, after adjustment, the RRs were 0.78 (95% CI, 0.61–1.01) for total stroke and 0.74 (95% CI, 0.54–1.02) for ischemic stroke.

In conclusion, there are few studies to date that specifically examine the relationship between wholegrain intakes and risk of stroke. The studies described above have shown mixed results when adjustment has been made for potential confounders. However, the trends are strongly suggestive of a protective effect of wholegrain on risk of stroke.

### 3.4. Cereal Fiber and CHD

A number of researchers have found that a higher intake of cereal fiber is associated with a lower risk of CHD, although results tend not to be statistically significant after adjusting for multiple confounding factors [51,53,60–62]. Pereira *et al*. [63] pooled the results of nine studies, yielding a total of 310,278 men and women who had a total of 6959 CHD events, of which 1869 were deaths. With cereal fiber fitted as a continuous variable, each 10 g/day increment in cereal fiber had a non-significant relative risk of 0.90 for total CHD events and a significant relative risk of 0.75 for CHD deaths. The associations appeared to be independent of other dietary factors, sex, age, baseline BMI, history of hypertension, diabetes and hypercholesterolemia. The authors suggest that failure to reach significance for total CHD events may be because of the limitations of some FFQs to accurately quantify cereal fiber intake or to small numbers of events in women.

Anderson *et al.* [53] also performed a pooled analysis that explored the relationship between wholegrains and whole wheat bread, cereal fiber, total dietary fiber, fruits and vegetables and risk for CHD. After adjustment for confounding factors, they found that the strongest inverse association was between wholegrain and whole wheat bread intake and risk of CHD. Cereal fiber by itself had the least influence on CHD risk (RR 0.90; 95% CI, 0.80–1.01). A similar finding was made by Mozaffarian *et al.* [60]. After adjustment for potential confounders, the overall reduction in CVD risk from consumption of cereal fiber was 14% (HR 0.86, 95% CI, 0.075–0.99) when the 20th percentile of intake (1.7 g/day) was compared with the highest quintile (46.3 g/day). However, when post hoc analyses were conducted to investigate whether the observed lower CVD risk was related to fiber from any specific food group, it was found that the lower risk appeared to be predominantly related to fiber intake from dark breads such as whole wheat, rye or pumpernickel (*i.e*., wholegrain intake) (HR 0.76; 95% CI, 0.64–0.90). Other cereal fibers had a non significant influence (high fiber, bran or granola cereals (HR 0.99; 95% CI, 0.84–1.17), other cold cereals (HR 0.98; 95% CI, 0.94–1.02), cooked cereals (HR 1.01, 95% CI, 0.92–1.11)). The authors did not state whether these other cold or cooked cereal fibers were wholegrain or refined grain products.

In contrast, Jensen *et al.* [57] investigated the association between daily intake of wholegrains, additional bran and germ and CHD risk in men in a cohort of 42,850 health professionals. As described earlier, they found that men with the highest intake of wholegrain had an 18% lower risk of CHD than men in the lowest quintile. To ascertain whether the effect of added bran and germ conferred even greater benefits, the wholegrain content of all grain foods eaten was determined, and bran or germ contained in those foods measured as an amount that would typically be found in the type of wholegrain. Wheat bran, corn bran, oat bran, rice bran and wheat germ added to foods either during processing or by participants while cooking were considered to be added bran and germ. They found that the HR of CHD in men with the highest intake of added bran (median intake 11.10 g/day) was 0.70 (95% CI, 0.60–0.82) compared with men with no intake of added bran. Thus, men with the highest intake of added bran had a 30% lower risk of CHD than men who do not consume added bran. Added germ was not associated with CHD risk (although the intake of added germ was very low in this population).

Bazzano *et al.* [62] examined the relationship between dietary fiber intake and risk of CHD and CVD in 9776 adults who participated in the First National Health and Nutrition Examination Survey (NHANES 1) Epidemiologic Follow-up Study (NHEFS) conducted in the US. After adjusting for ‘healthy habits’ (regular exercise, not smoking, low dietary intake of cholesterol and saturated fats), they found that consumption of at least 4.5 g of cereal fiber per 1735 kcal was associated with a non significant 20% lower risk of CHD (RR 0.80, 95% CI, 0.63–1.01) and a non significant 11% lower risk of death from CHD (RR, 0.89, 95% CI, 0.76–1.02). A limitation of this study is that DF was estimated using a single 24-h dietary recall with no follow-up data collected, which may have resulted in misclassification of usual DF intake (there is no data to show which is the best instrument to assess fiber intake).

Lairon *et al.* [64] investigated the relationship between DF intake and cardiovascular risk factors in a subsample of 4.080 people (2168 male, 1912 women; 45–60 years at inclusion) drawn from the Supplementation en Vitamines et Mineraux Antioxydants (SU.VI.MAX) study population of 12,735 participants. This study was a randomized double-blind, placebo-controlled primary prevention trial designed to test the efficacy of daily supplementation with antioxidant vitamins and minerals at non-pharmacological doses in reducing cancers and CVD. The study commenced in 1994 and followed subjects for 8 years. Total DF intake data were ascertained by obtaining 12 different, validated 24-h dietary records from each participant during the 4 years after inclusion in the study. The dietary questionnaire came with instruction manuals for coding foods, including photographs for selecting seven different portion sizes. A number of clinical and biochemical indices were determined when participants joined the study, and preliminary data on the association of these indices with level and type of DF intake was presented. For men, a number of significant differences were found between the participants in the highest and lowest quintiles of total DF intake. Cereal fiber was most consistently associated with the studied variables, and the highest intake was significantly associated with a lower BMI, systolic blood pressure and plasma glucose. For women, the only significant difference between those participants with the highest *vs* the lowest quintile of total dietary fiber intake was observed for a lower BMI in those with the highest intake. For women, only vegetable fiber displayed an association. To date, detailed clinical data and the incidence of cardiovascular events in relation to fiber intake from this study have not been published.

Ness *et al.* [65] followed up a secondary prevention, randomized controlled trial in South Wales, UK, conducted by Burr *et al.* [66]. Between 1983 and 1987, Burr *et al*. [66] allocated men under 70 years who had survived a myocardial infarction to receive fiber advice or not (other groups received fish advice/or not and fat advice/or not). Participants were encouraged to eat at least six slices of wholemeal bread per day, or an equivalent amount of cereal fiber from a mixture of wholemeal bread, high-fiber breakfast cereals and wheat bran. At 6 months, cereal fiber intake in the fiber advice group was 19 and 9 g/day in those not given fiber advice. At 2 years, fiber intake was 17 and 9 g/day, respectively. After 2 years, the results were unclear, in that total mortality increased (not statistically significant) in those who were advised to eat more wholegrain. There were a number of limitations to this study including compliance, short follow-up and no objective check of fiber intake. When Ness *et al.* [51] followed up (between 1999 and 2000) the surviving participants from this cohort, they found no differences in self-reported weight, current smoking, medication use, aspirin use or dietary supplement use. At follow-up, those allocated to receive fiber advice had a significantly higher fiber intake, although the absolute difference was small. CHD mortality increased in those given fiber advice in the first two follow-up time periods, but reduced in the later time periods such that there was no long-term effect on survival. In following up Burr *et al.*’s cohort, Ness and co-workers acknowledge that dietary advice stopped after 2 years and recent data were collected when around half of the original participants had died. The researchers cannot discount the possibility that the diets of those who survived are different from those who did not. Follow-up diet was assessed with a limited number of questions focused on fiber intake; questionnaire data were not collected on other aspects of current diet and it is thus possible that important differences in diet were not detected. The authors concluded that the failure of longer term data to confirm the initial but non-significant increased risk of CHD deaths in men who were given advice to eat more cereal fiber, suggested that increased fiber intake was not harmful. The results do, however, suggest that increasing cereal fiber intake by a small degree does not confer any immediate survival advantage. It must be borne in mind, however, that this was a secondary prevention trial of CHD, in which the influence of environmental factors on the pathological process is likely to be weaker than in a primary prevention trial [51].

Jacobs *et al*. [67] argued that the potential health effects of cereal fiber may depend on its source. They hypothesized that the nutrients eaten with fiber (antioxidants and minerals) play a role in protection against chronic disease and that the results of studies of cereal fiber intake would depend on the mix of fiber from wholegrain compared to refined grain. They examined all-cause, CHD and cancer mortality data from 11,040 participants involved in the prospective Iowa Women’s Health Study. They compared two groups of women, matched to consume B6 g/day of total cereal fiber. One group consumed on average 77% of their cereal fiber from refined grain sources. The second group consumed on average 71% of their grain fiber from wholegrain sources. This group was followed from baseline in 1986 to December 1997, during which time 1341 total deaths occurred (274 from CHD; 247 with complete data included in multivariate analysis). After adjusting for healthy lifestyle characteristics, a multivariate regression showed that women who consumed on average 1.9 g refined grain fiber/2000 kcal and 4.7 g wholegrain fiber/2000 kcal had a 17% lower all-cause mortality rate (95% CI, 0.73–0.94) and a non significant 11% lower CHD rate (95% CI, 0.66–1.20) than women who consumed predominantly refined grain fiber (4.5 g/2000 kcal but only 1.3 g wholegrain fiber/2000 kcal). Despite these findings reflecting statistically non significant reductions for CHD deaths, the authors state that women who were excluded from the matched design because they consumed more than 6 g/2000 kcal of wholegrain fiber (generally accompanied by small amounts of refined grain fiber) had statistically significantly reduced total, CHD and all-cause mortality rates, compared to women who ate little wholegrain fiber.

These results highlight the emerging ‘wholegrain story’ which argues that health benefits stem from more than just the fiber; the wholegrain is nutritionally more important because it delivers a whole package of nutrients and phyto-protective substances that may work synergistically to promote health [53]. Thus although fiber is an important component of wholegrains, many studies have not shown an independent effect of fiber alone on CHD events and/or deaths. Thus in terms of CHD prevention, fiber is probably best obtained from wholegrain sources.

### 3.5. Soluble Fiber and CHD

There is little epidemiological data on the association between soluble fiber and CHD even though soluble fiber clearly lowers serum cholesterol concentration and also lowers glucose in diabetics. A cholesterol-lowering claim is allowed for oat-derived beta glucan (rolled oats, oat bran, whole oat flour, the soluble fraction of alpha-amylasehydrolysed oat bran or whole oat flour with a beta-glucan content up to 10 percent) by the United States Food and Drug Administration (USFDA, 1997).

In a study of female health professionals, fiber was associated with protection from heart disease [68]. The association with insoluble fiber was stronger than for soluble fiber although neither separately was significant. The association with total fiber was insignificant after full adjustment.

In a secondary prevention study in men with angina, Burr *et al*. [69] found no effects of advice to increase fruit and vegetables and oats on total or cardiovascular mortality although compliance was not well assessed and, in the small number of people actually assessed, it was low.

A meta-analysis of eleven clinical intervention trials [70] involving legumes (other than soy beans) found overall a 6.2% lowering of LDL-C and 22% lowering of triglycerides. The hypocholesterolemic effects of legumes appear related, in estimated order of importance, to soluble dietary fiber, vegetable protein, oligosaccharides, isoflavones, phospholipids and fatty acids, and saponins. Oat beta glucan may be ineffective at lowering serum cholesterol concentration when baked into bread and cookies. Jenkins *et al*. [71] confirmed that 4 servings/day of beta glucan or psyllium delivering 8 g/day of soluble fiber lowered total cholesterol by 2.1% which would have a useful population rather than individual effect on heart disease risk. In conclusion, soluble fiber clearly lowers cholesterol to a small and significant degree and one would expect that this would reduce CHD events.

## 4. Dietary Fibers and Metabolic Diseases

Furthermore, two other well-known cardiovascular risk factors (type 2 diabetes and obesity) seem to be influenced by intake of DF. Vegetarians who consume a high-fiber lacto-ovo vegetarian diet appear to have a lower risk of mortality from diabetes-related causes compared to nonvegetarians [72]. Consumption of wholegrain cereals is associated with a lower risk of diabetes. Importantly, the same dietary pattern appears to lower the risk of obesity, itself an independent risk factor in the etiology of type 2 diabetes. A report showed that in 91,249 women questioned about dietary habits in 1991, greater cereal fiber intake was significantly related to lowered risk of type 2 diabetes. It can be hypothesized that a reduction in the general and postprandial glycemic and insulinemic response may delay the development of insulin resistance and thus the development of diabetes [73]. Much of the research in this area has studied the effect of DF on the management rather than the prevention or etiology of diabetes. There is good evidence that diminished glucose absorption lowers the insulin response to a meal [74]. It seems that soluble fiber may play a role because large amounts of soluble DF have been shown to reduce postprandial glucose concentration and insulinemic responses after a single meal in both normal and diabetic subjects. However, the effect appears to be dependent on viscosity rather than on solubility per se. The very viscous gum, guar gum, gum tragacanth, and oat gum are all very effective whereas psyllium and some pectins are less viscous and less effective. Glucose is trapped in the gel matrix, such that there is less movement toward the absorptive brush border of the surface of the intestinal wall. However, recent large prospective cohort studies showed that is mainly the consumption of insoluble cereal DF and wholegrains that is consistently associated with reduced risk of type 2 diabetes [75–77].

A number of recent studies give novel insights that might help establish a metabolic link between insoluble DF consumption and reduced diabetes risk. Potential candidates are improved insulin sensitivity and the modulation of inflammatory markers, as well as direct and indirect influences on the gut microbiota [78]. A breakthrough recent paper published reported that microbial populations in the gut are different between obese and lean people, and that when the obese people lost weight their microflora reverted back to that observed in a lean person, suggesting that obesity and type 2 diabetes may have a microbial component. The levels of glucose tolerance or severity of diabetes should be considered while linking microbiota with obesity and other metabolic diseases in humans [79–81].

Currently, the American Diabetes Association recommends that diabetic patients consume 14 g/1000 kcal/day of fiber because a high amount of fiber is necessary to improve glycemic control. This amount is between 2 and 3 times higher than that consumed by individuals in many developed countries [82].

The association between fiber intake and obesity or CHD is confirmed by several epidemiological studies. DF can modulate body weight by various mechanisms. Fiber-rich foods usually have lower energy content, which contributes to a decrease into the energy density of the diet [83]. Foods rich in fiber need to be chewed longer, leading to an increase in the time needed to eat the food and in the feeling of satiety. The fibers which make up viscous solutions also delay the passage of food from the stomach to duodenum and contribute to an increase in satiety and a decrease in energy consumption [84]. In the intestine, the incorporation of fiber may complicate the union between digestive enzymes and their substrate, thus slowing down the absorption of nutrients [85]. It is also important to note that the effects of DF consumption on body weight may be related to different gut hormones which regulate satiety, energy intake and/or pancreatic functions [86].

Alfieri *et al.* [87] assessed the total fiber intake by means of 3-day food records in 3 population groups (one normal weight group, with a BMI between 20 and 27.0 kg/m^2^; one moderately obese group, with a BMI between 27.1 and 40 g/m^2^; and one severely obese group, with a BMI > 40.0 kg/m^2^). These authors showed that fiber intake was significantly higher in the normal weight group and was inversely associated with BMI after adjusting for several potential confounders (sex, age, education level and income). The CARDIA (Coronary Artery Risk Development in Young Adults) study, a multicenter population-based cohort study carried out over 10 years, examined 2909 young individuals to determine the relationship between total DF intake and plasma insulin concentrations, weight and other CVD risk factors. After adjusting for BMI and multiple dietary (total energy, fat, alcohol intake) and potential non-dietary confounders (gender, education, physical activity, basal body weight, tobacco use), the study reported an inverse association between total fiber intake, plasma insulin concentrations and body weight gain [88] suggesting that fiber may play an important role in the prevention of insulin resistance and obesity.

## 5. Summary

Results from all epidemiologic studies described above underline that the intake of wholegrain foods clearly protects against CHD and stroke but the exact mechanism is not yet clear. Moreover, the intake of high carbohydrates (from both grain and non-grain sources) in large amounts is associated with an increased risk of CHD in overweight and obese women even when fiber intake is high, but this requires further confirmation in normal-weight women.
